# The effects of aromatherapy on anxiety and sleep quality in maternal women: a systematic review and meta-analysis

**DOI:** 10.3389/fpubh.2025.1701126

**Published:** 2025-11-28

**Authors:** Ziwen Wang, Chuangui Mao, Sihang Zeng, Lunxin Chen, Zhiyong Feng, Weiguo Liu

**Affiliations:** 1 College of Physical Education and Health, Guangxi Normal University, Guilin, China; 2 School of Physical Education and Sports, Central China Normal University, Wuhan, China

**Keywords:** aromatherapy, anxiety, sleep quality, mothers, meta-analysis

## Abstract

**Objective:**

This study aims to conduct a systematic review of the efficacy of aromatherapy in treating maternal anxiety and improving sleep quality during pregnancy and postpartum, thereby providing scientific evidence for clinical practice.

**Methods:**

We searched databases including PubMed, Cochrane, Web of Science, and Embase from their inception to October 2025, identifying 13 eligible studies. The methodological quality of included studies was assessed using the Cochrane Risk of Bias Tool. Meta-analysis was performed using Stata 15.0.

**Results:**

Aromatherapy improved anxiety levels [SMD = −0.4, 95% CI (−0.68, −0.14), *p* = 0.002, *I*^2^ = 63.8] and enhanced sleep quality [SMD = −0.59, 95% CI (−0.98, −0.21), *p* = 0.002, *I*^2^ = 88.7]. Subgroup analysis revealed that prenatal aromatherapy effectively reduced anxiety [SMD = −0.75, 95% CI (−0.97, −0.52), *p* = 0.000, *I*^2^ = 0] and improve sleep quality [SMD = −1.18, 95% CI (−2.08, −0.29), *p* = 0.000, *I*^2^ = 90.6].

**Conclusion:**

This meta-analysis demonstrates the positive effects of aromatherapy in improving maternal anxiety and sleep quality, and identifies effective intervention phases. However, due to the limited number of included studies, future research should incorporate more high-quality studies to further consolidate and validate the reliability of these findings.

**Systematic Review Registration:**

PROSPERO, NO. 2025 CRD420251063171. https://www.crd.york.ac.uk/PROSPERO/view/CRD420251063171.

## Introduction

In recent years, with societal development, there has been increasing attention on the mental health of pregnant women and new mothers (1). Hormonal fluctuations occur during pregnancy and postpartum periods, potentially leading to emotional instability, anxiety, and often poor sleep quality (2, 3). Data indicates that the incidence of anxiety during prenatal and postpartum periods ranges from approximately 16% to 25% (4, 5), while insomnia affects 43.9% to 67.8% of these individuals (6, 7). These psychological issues severely jeopardize the physical and mental health of both mothers and infants, potentially triggering delusions, psychotic episodes, and even suicide among mothers (8, 9). Consequently, developing effective interventions for maternal anxiety and sleep disorders has become an urgent public health priority.

Currently, approaches to alleviating maternal anxiety and sleep disorders primarily include pharmacological and non-pharmacological treatments. Since medication often carries side effects that may adversely affect both infants and mothers themselves (10), expectant and new mothers should exercise caution with drug therapies and prioritize non-pharmacological interventions. Among non-pharmacological methods, aromatherapy stands out for its low cost, simplicity, eco-friendly nature, and significant efficacy. Over the years, it has gained widespread acceptance in numerous countries and is extensively used to improve symptoms such as insomnia and anxiety (11). Also known as essential oil therapy, aromatherapy is a common non-pharmacological approach. It utilizes aromatic essences extracted from natural plants, delivered through oral ingestion, inhalation via aromatherapy, massage, or bathing, to penetrate the body and produce pharmacological effects (12–17). When taken orally, essential oil components can enter the bloodstream (12); given their lipophilic nature, they can also be easily delivered to organs throughout the body via topical application and massage (12). Additionally, in inhalation aromatherapy, essential oil-containing vapors enter the circulatory system not only through the nasal capillary network and pulmonary bronchi but also directly stimulate specific brain regions via the olfactory epithelium (12, 16). The mechanism by which essential oils trigger effects within the brain via the olfactory system involves stimulation of olfactory receptor cells in the nasal epithelium connected to the olfactory bulb. Signals are transmitted through the olfactory bulb and tract to the brain's limbic system and hypothalamus. Upon reaching the olfactory cortex, these signals trigger neurotransmitter release, thereby producing the mood-regulating effects associated with essential oil use (18–20).

Although numerous scholars have conducted extensive research on improving maternal mental health, meta-analyses examining aromatherapy's effects on reducing maternal anxiety and enhancing sleep quality remain relatively scarce. One meta-analysis demonstrated that lavender essential oil significantly improves postpartum sleep quality (21). However, it included only three studies, resulting in low statistical power. Furthermore, lavender essential oil is just one type of essential oil used in aromatherapy, each with potentially distinct therapeutic properties due to their unique volatile constituents. Therefore, findings based solely on lavender oil cannot be generalized to determine the efficacy of aromatherapy as a whole, which encompasses a diverse range of essential oils. Additionally, no meta-analysis has yet explored the efficacy of aromatherapy for postpartum anxiety. Consequently, there remains a lack of systematic evaluation evidence regarding the precise therapeutic effects of aromatherapy on improving maternal anxiety symptoms and sleep quality.

In summary, this study proposes to employ a meta-analysis approach to comprehensively evaluate the effects of aromatherapy interventions on maternal anxiety and sleep quality, aiming to provide evidence-based medical support for clinical practice. Outcome measures include the Depression Anxiety Stress Scales-21 (DASS-21) scale, State-Trait Anxiety Inventory (STAI) scale, and Pittsburgh Sleep Quality Index (PSQI) scale.

## Methods

### Protocol and registration

This study adheres to the Preferred Reporting Items for Systematic Reviews and Meta-Analyses (PRISMA) guidelines and was registered in the International Prospective Registration Database for Systematic Reviews (PROSPERO) on May 29, 2025, with registration number CRD420251063171.

### Eligibility criteria

This systematic review and meta-analysis adheres to the PRISMA statement and establishes inclusion and exclusion criteria based on the PICOS framework. Specific criteria for study inclusion and exclusion are detailed in Table 1.

**Table 1:** Information sources.

**Category**	**Inclusion criteria**	**Exclusion criteria**
Research Subjects	Pregnant women and postpartum women	Pregnant women and postpartum women with significant physiological disorders
Intervention measures	All received aromatherapy	Did not receive aromatherapy or received multiple treatments
Control measures	Placebo or purified water	Drug the rapy, routine care, no intervention, etc.
Outcome	Scale measuring participants' anxiety and sleep quality	Research data unavailable (e.g., mean ± standard deviation for pre- and post-tests); despite communication with the corresponding author, full-text access to the study remains unavailable
Research design	Randomized controlled trial	Animal studies, Case studies, reviews, Cross-over trials, Patents, Registration agreements

### Information sources

Computer-based searches were conducted in publicly available English-language literature databases, including Cochrane, Embase, PubMed, and Web of Science. The search period spanned from the inception of each database up to October 30, 2025.

### Search strategy

Searches were conducted in databases using Boolean operators (such as “OR” and “AND”) in combination with a series of keywords. The retrieval terms included Pregnancy, Postpartum Period, Aromatherapy, Aroma Oil, Sleep Quality, Anxiety, etc. The detailed search strategy for the PubMed database is presented in Table 2, while the search strategies for the remaining databases are outlined in Appendix 1.

**Table 2:** PubMed search strategy information.

**No**.	**Search strategy**	**Start and end dates**
#1	“Pregnancy”[MeSH Terms]	From the establishment of the database to October 30, 2025
#2	“maternal”[Title/Abstract] OR “pregnant”[Title/Abstract] OR “gestational”[Title/Abstract] OR “pregnancy”[Title/Abstract]	
#3	“Postpartum Period”[MeSH Terms]	
#4	“postpartum”[Title/Abstract] OR “post-pregnancy”[Title/Abstract]	
#5	“Aromatherapy”[MeSH Terms]	
#6	“Aroma”[Title/Abstract] OR “aroma oil”[Title/Abstract] OR “essential oil”[Title/Abstract] OR “Aromatherapies”[Title/Abstract] OR “aroma therapy”[Title/Abstract] OR “aroma therapies”[Title/Abstract]	
#7	“Sleep Quality”[MeSH Terms]	
#8	“Insomnia”[Title/Abstract] OR “sleep disorder”[Title/Abstract] OR “sleep wake disorders”[Title/Abstract] OR “sleep disturbance”[Title/Abstract] OR “sleep problem”[Title/Abstract]	
#9	“Anxiety”[MeSH Terms]	
#10	“Anxious”[Title/Abstract] OR “Nervousness”[Title/Abstract] OR “Anxiousness”[Title/Abstract]	
#11	#1 OR #2	
#12	#3 OR #4	
#13	#5 OR #6	
#14	#7 OR #8	
#15	#9 OR #10	
#16	#11 OR #12	
#17	#14 OR #15	
#18	#13 AND #16 AND #17	

### Selection process

**Figure 1:**
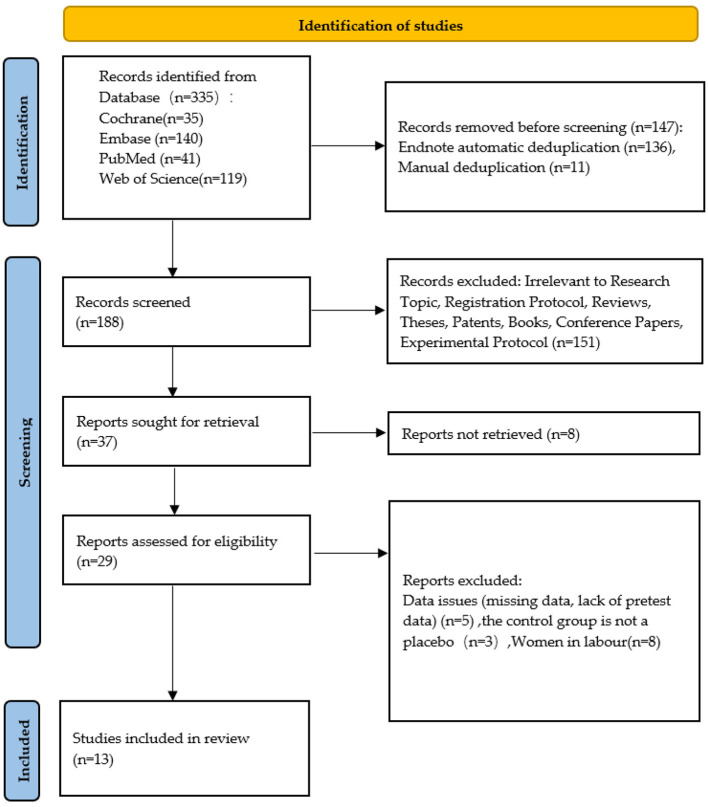
Literature screening flowchart.

First, researchers imported the retrieved studies into the EndNote X9.1 (Version X9; Thomson ResearchSoft, USA) document management system to remove duplicates. Subsequently, we excluded obviously ineligible literature by reviewing titles and abstracts, and determined the final included studies through full-text reading, group discussions, and contacting authors for additional research details. The final list of included studies was converted to Microsoft Excel. Information retrieval and literature screening were conducted independently by two research members (ZW and CM). Finally, a third research member (LC) resolved discrepancies and verified the final included studies. The study selection process is summarized in the PRISMA flow diagram (Figure 1).

### Data collection process

A standardized electronic data extraction form was developed based on the study protocol. Two co-authors (ZW and SZ) independently extracted data from each included study. Prior to comprehensive data extraction, the form was pilot-tested on samples from three included studies, with refinements made to enhance clarity and completeness. From each included study, we extracted the following information using the data extraction form: (1) Study characteristics (first author, publication year); (2) Participant characteristics (sample size, age, intervention phase); (3) Intervention details (method, duration, frequency, and cycle of interventions for both experimental and control groups); (4) Outcome measures; (5) Information relevant to risk of bias assessment. After independent extraction, the two reviewers compared their respective results and resolved discrepancies through discussion. Remaining disagreements were adjudicated by a third co-author (WL). For studies reporting ambiguous or incomplete data (e.g., missing standard deviations, poorly defined outcomes), we attempted to contact the original authors via email (at least twice, spaced 2 weeks apart) to request missing data. For studies reporting standard deviations (standard errors) and means graphically, data were extracted using the GetData graph digitizer software (http://www.getdata-graph-digitizer.com/) (22).

### Data items

The outcomes included in this systematic review and meta-analysis are shown in Table 3.

**Table 3:** Outcomes included in the study.

**Measurement metrics**	**Scale**
Anxiety	State-Trait Anxiety Inventory (STAI);State-Trait Anxiety Inventory Simplified Version (STAI-6): Depression Anxiety Stress Scales-21 (DASS-21);
Sleep quality	Pittsburgh Sleep Quality Index (PSQI)

### Study risk of bias assessment

Risk of bias was assessed using the Randomized Controlled Trials Risk of Bias Assessment Tool 2.0 (Rob 2.0), which primarily includes the following five assessment indicators: (1) bias arising from randomization processes; (2) bias arising from deviation from the intended intervention; (3) bias arising from missing outcome data; (4) bias arising from outcome measurement; (5)bias from selective reporting of results (23). Each indicator comprised 3–7 signal questions, with the RCT risk of bias assessment tool 2.0 automatically generating the quality grade for included studies. Risk of bias was assessed by two researchers (ZW and SZ). In cases of substantial disagreement, a third researcher (ZF) was consulted to resolve the discrepancy.

### Effect measures and synthesis methods

This study employed Stata 15 software (StataCorp. 2017. Stata Statistical Software: Release 15. College Station, TX: StataCorp LLC.) to conduct statistical analyses on the included data. Although the STAI scale, DASS-21 scale, and PSQI scale were respectively adopted as measures of anxiety levels and sleep quality in this study, due to variations across countries and versions, all results are presented using the Standard Mean Difference (SMD) and 95% confidence interval (CI), with a significance level of α = 0.05. The *I*^2^ statistic was used to assess study heterogeneity; an *I*^2^ < 25% was considered indicative of negligible heterogeneity, an *I*^2^ between 25% and 75% indicated moderate heterogeneity, and an *I*^2^ > 75% signified substantial heterogeneity (24). Although the Cochrane Handbook does not provide universal recommendations for model selection, all effects in this study were analyzed using a random-effects model due to the diversity of study participants and variability in intervention methods, and to avoid potential underestimation of heterogeneity by fixed-effects models (25). *p* < 0.05 was considered statistically significant. When heterogeneity was present, subgroup analyses or stepwise exclusion methods were employed to identify sources of heterogeneity.

### Reporting bias assessment

This study assessed the risk of publication bias using funnel plots and Egger's test. A *P*-value > 0.05 indicates no publication bias. If publication bias was detected, missing studies were estimated and supplemented using imputation methods. Sensitivity analysis was performed to evaluate the robustness of the results.

### Certainty assessment

Two researchers (ZW and CM) assessed evidence quality using the GRADE approach, completed via the GRADEpro GDT online tool. Following the GRADE framework, evidence quality was systematically evaluated across five dimensions: risk of bias, inconsistency, indirectness, imprecision, and publication bias (26)]. For each outcome, evidence quality was rated as high, moderate, low, or very low based on a comprehensive assessment of confidence in the effect estimates (27). Any disagreements during the assessment process were resolved through consensus.

## Results

### Study selection

The database search yielded 335 records. Subsequently, EndNote automatically removed 136 duplicate records, and 11 duplicate records were manually deleted, totaling 147 records removed. Preliminary screening based on titles and abstracts identified 188 unique records. Among these, 151 records were excluded due to clear non-compliance with criteria (e.g., review studies, animal experiments, irrelevance to the research topic). Full texts were sought for the remaining 37 records. Despite contacting authors and libraries, full texts could not be obtained for 8 records. Researchers conducted eligibility assessments on 29 full-text articles. Sixteen articles were excluded for the following reasons: missing experimental data (5 articles), control groups not being placebo-controlled (3 articles), and interventions during the delivery process (8 articles). Consequently, 13 studies met the inclusion criteria and were incorporated into the systematic review and meta-analysis. The detailed screening process is outlined in the PRISMA flow diagram (Figure 1).

### Study characteristics

This systematic review and meta-analysis included 13 studies involving 1,042 participants, investigating the effects of aromatherapy on maternal anxiety and sleep. All participants in this study were pregnant women or new mothers, and all received aromatherapy treatment. Specific details are presented in Table 4.

**Table 4:** Basic characteristics of the literature included.

**Author**	**Inter-vention period**	**Experimental group**					**Control group**					**Testing Period**	**Outcome Indicator**
		**Sample size/age**	**Intervention measures**	**Intervention time**	**Frequency**	**Period**	**Sample size/age**	**Intervention measures**	**Inter-vention time**	**Frequency**	**Period**		
1. Afshar (38)	Postnatal interventions	79/28.06 ± 4.105	Lavender Essential Oil Inhalation	All night	Four times a week	8 weeks	79/28.14 ± 4.128	Placebo inhalation	All night	Four times a week	8 weeks	4 weeks, 8 weeks	PSQI
2. Effati-Daryani (39)	Prenatal intervention	47/28.2 ± 4.99	Lavender Cream Apply to the legs.	Apply before bedtime until it wears off.	Once a day	8 weeks	47/28.1 /ks.9	Placebo cream applied to the legs	Apply before bedtime until it wears off.	Once a day	8 weeks	4 weeks, 8 weeks	DASS-21
3. Mirghafourvand (40)	Postnatal interventions	48/28.8 ± 5.3	orange peel essential oil drink	–	Three times a day	8 weeks	48/28.1 ± 6.3	Placebo drink	–	Three times a day	8 weeks	8 weeks	PSQI
4. Mirghafourvand (41)	Postnatal interventions	48/28.8 ± 5.3	Orange peel essential oil drink	–	Three times a day	8 weeks	48/28.1 ± 6.3	Placebo drink	–	Three times a day	8 weeks	8 weeks	STAI
5. Effati-Daryani (42)	Prenatal intervention/ Postnatal interventions	47/28.2 ± 5.0	Lavender cream Apply to the skin on your legs	Apply before bedtime until it wears off.	Once a day	6 weeks	45/28.2 ± 5.0	Placebo cream applied to the skin on the legs	Apply before bedtime until it wears off.	Once a day	6 weeks	4 weeks, 6 weeks	PSQI
6. Amzajerdi (43)	Prenatal intervention	33/26.97 ± 4.57	Mint aroma group Inhale	20 min	Twice a day	1 week	33/28.4 ± 3.91	Placebo group Inhale	20 min	Twice a day	1 week	1 week	STAI
7. Abbasijahromi (44)	Postnatal interventions	30/18–35 year-old	A: Lavender essential oil Inhale B: Damask rose essential oils Inhale	30 min	One-time	One-time	30/18–35 year-old	Distilled water Inhale	30 min	One-time	One-time	After treatment	STAI
8. Burgess (45)	Postnatal interventions	24/29.7 ± 5.8	Lavender tabs Inhale	1–2 h	One-time	One-time	23/29.5 ± 3.8	Placebo tabs Inhale	1–2 h	One-time	One-time	After treatment	STAI-6
9. Chen (46)	Postnatal interventions	29/33.45 ± 4.36	Bergamot essential oil Inhale	15 min	Once a day	4 weeks	29/33.42 ± 4.21	Pure water Inhale	15 min	Once a day	4 weeks	2 weeks, 4 weeks	PSQI
10. Mohammadi (47)	Prenatal intervention	33/27.11 ± 4.55	Citrus aurantium essential oil Inhale	20 min	Twice a day	4 weeks	35/26.36 ± 4.89	Placebo essential oil Inhale	20 min	Twice a day	4 weeks	4 weeks	PSQI
11. Hossein (48)	Postnatal interventions	37/27.32 ± 5.41	Rose essence Inhale	All night	Once a day	4 weeks	37/26.00 ± 4.76	Distilled water Inhale	All night	Once a day	4 weeks	4 weeks, 12 weeks	PSQI
12. Nouira (49)	Postnatal interventions	50/31.3 ± 3.8	Lavender essential oil Inhale	30 min	One-time	One-time	50/32.3 ± 4.5	Distilled water Inhale	30 min	One-time	One-time	After treatment	STAI
13. Moradi (50)	Prenatal intervention	33/27.11 ± 4.55	Citrus Aurantium Inhale	20 min	Twice a day	4 weeks	35/26.36 ± 4.89	Odorless sweet almond oil Inhale	20 min	Twice a day	4 weeks	4 weeks	DASS-21

PSQI, Pittsburgh Sleep Quality Index; STAI, State-Trait Anxiety Inventory; STAI-6: State-Trait Anxiety Inventory Simplified Version; DASS-21; Depression Anxiety Stress Scales-21.

### Risk of bias in studies

**Figure 2:**
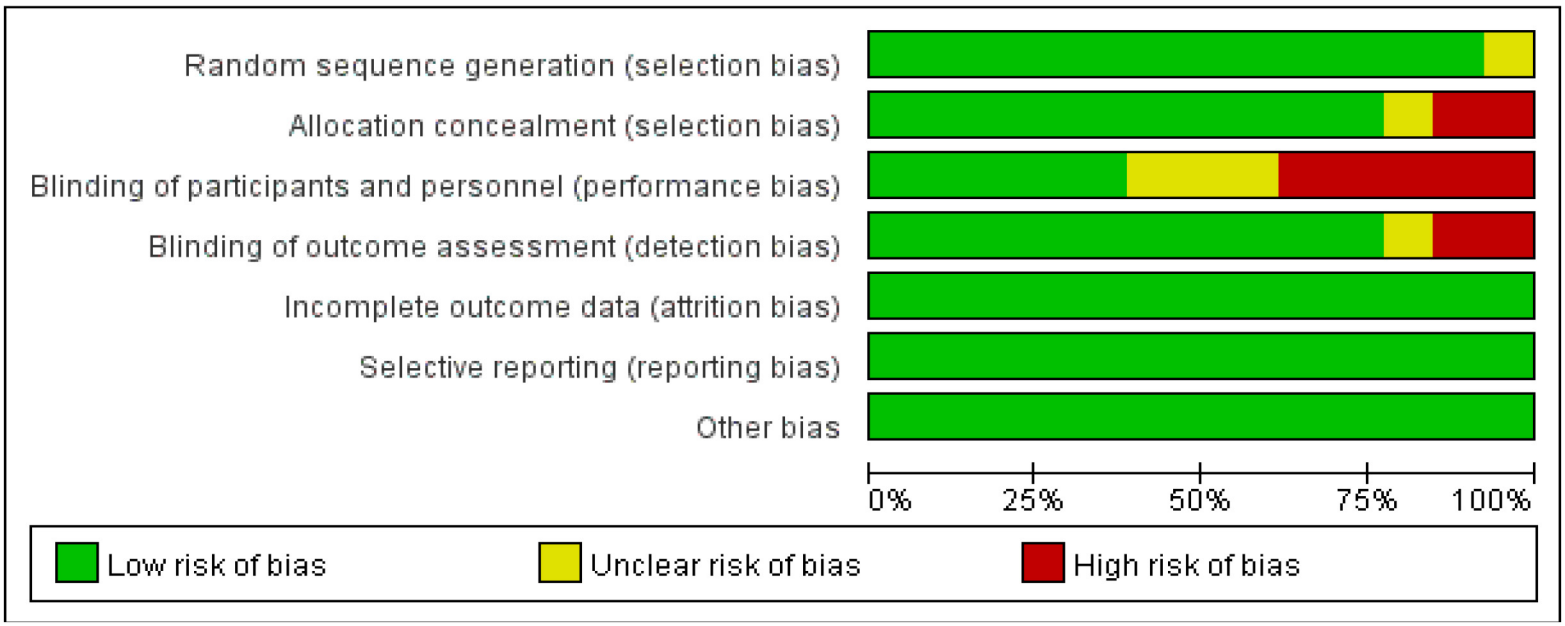
Risk of bias graph for all included studies.

**Figure 3:**
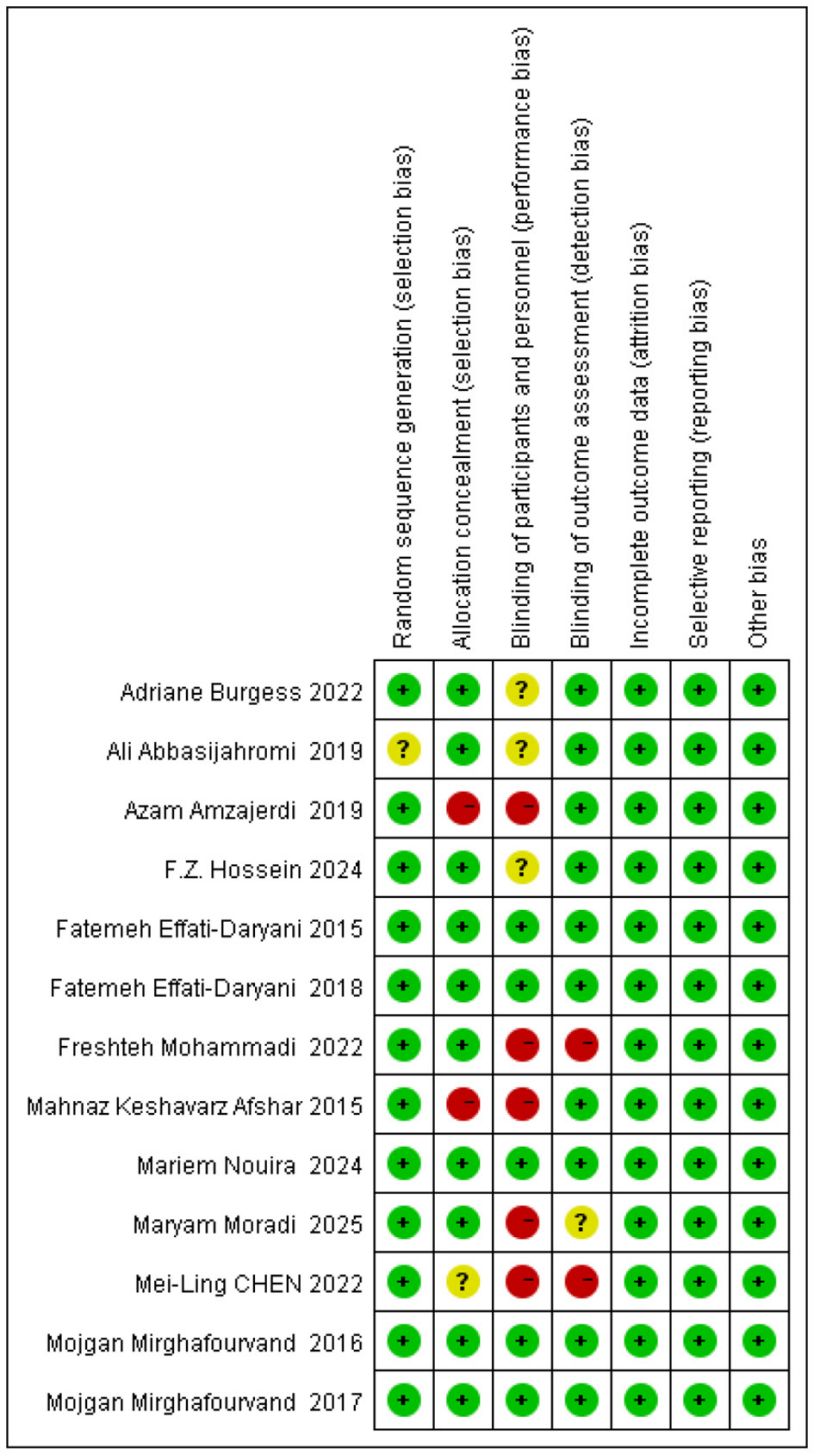
Risk of bias summary for all included studies.

Two researchers (A and B) assessed the quality of the included studies across seven domains using the Cochrane risk of bias tool. Regarding random allocation, one study had an uncertain risk of bias; for allocation concealment, eight studies had an uncertain risk of bias; concerning blinding of researchers and participants, one study had an uncertain risk of bias, while two studies had a high risk of bias. Regarding outcome assessment blinding, three studies had an uncertain risk of bias and four studies had a high risk of bias. All studies were at low risk for completeness of data reporting, selective reporting of results, and other biases (see Figures 2, 3).

### Reporting biases

**Figure 4:**
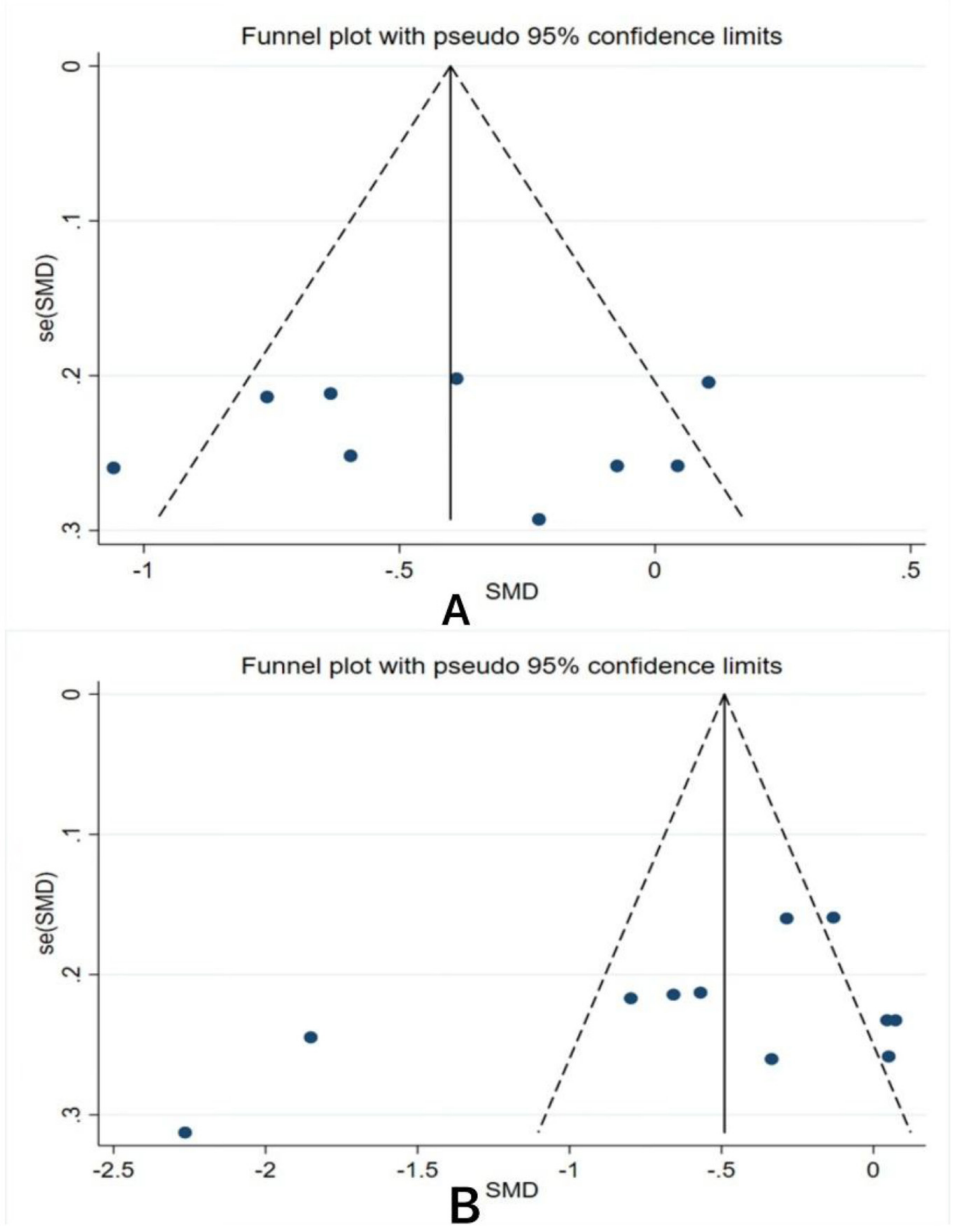
Funnel Chart Results. **(A)** Anxiety; **(B)** Sleep Quality.

This study conducted a publication bias test on the literature included for anxiety and sleep quality outcome measures. The funnel plot results indicate a potential risk of publication bias (Figure 4). The Egger test revealed no evidence of publication bias for anxiety (*t* < 0.001, *p* = 0.998) or sleep quality (*t* = −1.6, *p* = 0.144).

## Results of syntheses

### Sensitivity analysis

**Figure 5:**
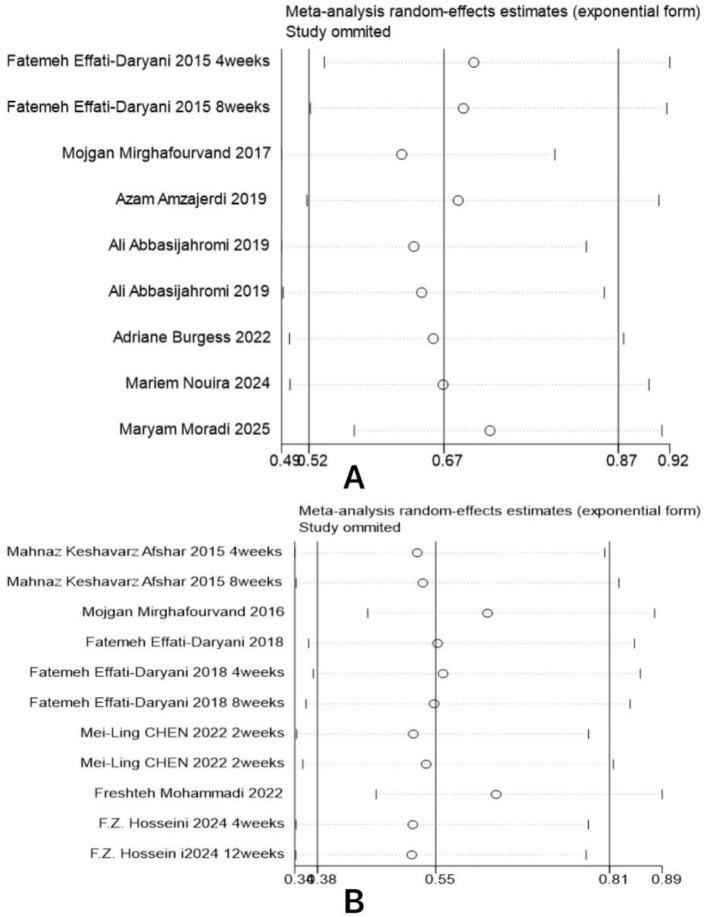
Sensitivity analysis results. **(A)** Anxiety; **(B)** Sleep Quality.

Sensitivity analysis for anxiety and sleep quality indicators was conducted by sequentially excluding studies. As shown in Figure 5, the point estimates of the pooled effect size remained within the 95% confidence interval (CI) of the overall pooled effect size after excluding any single study, indicating that the pooled results for these outcome measures are robust.

### The effect of aromatherapy on anxiety levels in pregnant and postpartum women

**Figure 6:**
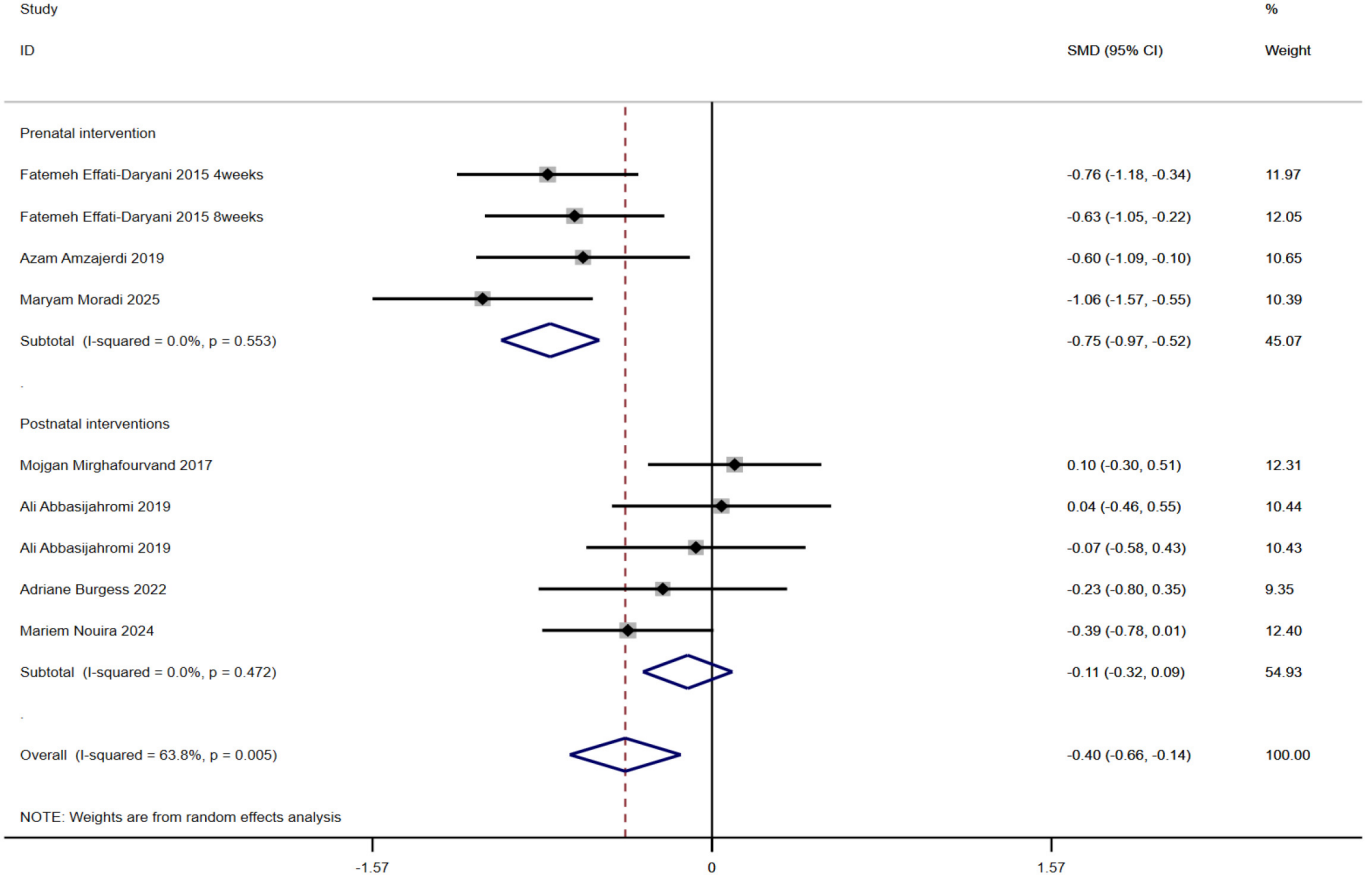
Anxiety forest plot.

A total of 7 studies involving 531 participants evaluated the efficacy of aromatherapy for maternal anxiety (Figure 6). Results indicated that aromatherapy significantly improved anxiety overall [SMD = −0.4, 95% CI (−0.68, −0.14), *p*= 0.002], with moderate heterogeneity (*I*^2^ = 63.8%, *p*= 0.005). Subgroup analysis revealed differing effects across intervention periods: prenatal intervention [SMD = −0.75, 95% CI (−0.97, −0.52), *p* = 0.000] showed no heterogeneity within the group (*I*^2^ = 0, *p* = 0.553). Postnatal interventions [SMD = −0.11, 95% CI (−0.32, 0.09), *p* = 0.278] showed no heterogeneity within groups (*I*^2^ = 0, *p* = 0.472).

### The effect of aromatherapy on sleep quality in pregnant and postpartum women

**Figure 7:**
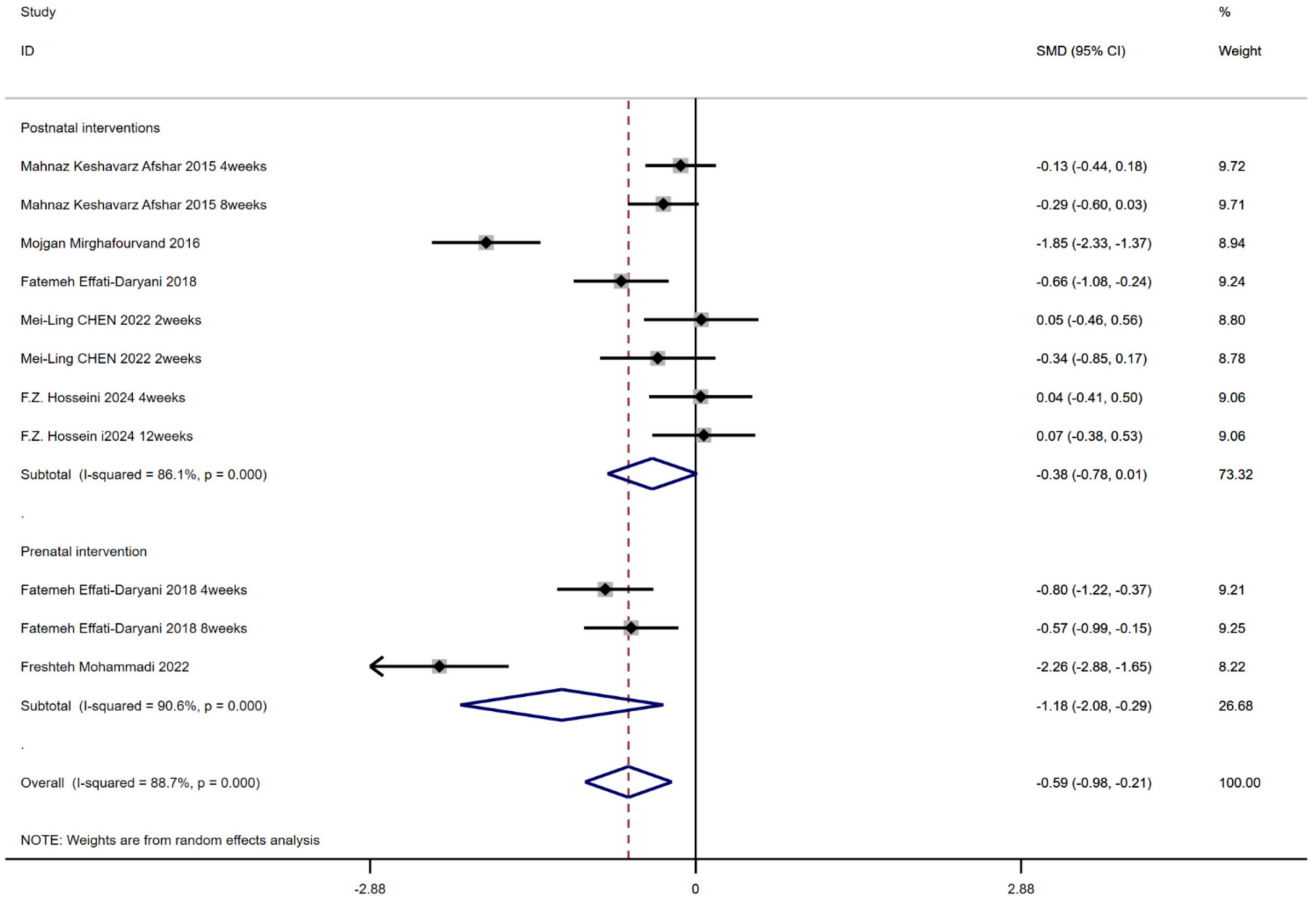
Sleep quality forest plot.

A total of 6 studies involving 577 participants evaluated the efficacy of aromatherapy on maternal sleep quality (Figure 7). Results indicated that aromatherapy significantly improved sleep quality overall [SMD = −0.59, 95% CI (−0.98, −0.21), *p* = 0.002], with high heterogeneity (*I*^2^ = 88.7%, *p* = 0.000). Subgroup analysis revealed differing effects across intervention periods: prenatal intervention [SMD = −1.18, 95% CI (−2.08, −0.29), *p* = 0.01] showed high heterogeneity within groups (*I*^2^ = 90.6, *p* = 0.000). Postnatal interventions [SMD = −0.38, 95% CI (−0.78, 0.01), *p* = 0.058] showed high heterogeneity within groups (*I*^2^ = 86.1, *p* = 0.000).

### Certainty of evidence

The GRADE tool was applied to rate the quality of evidence for outcome measures, with downgrading based on risk of bias, inconsistency, indirectness, imprecision, and publication bias in the original studies. Results indicate moderate-quality evidence for anxiety and sleep quality, as shown in Table 5.

**Table 5:** GRADE summary of evidence.

**Outcome**	**Number of studies**	**Study design**	**Risk of bias**	**Inconsistency**	**Indirectness**	**Imprecision**	**Confidence rating**
Anxiety	6	Randomized trials	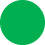	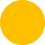	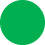	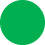	Moderate
Sleep quality	7	Randomized trials	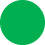	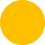	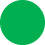	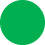	Moderate

## Discussion

The findings of this meta-analysis indicate that aromatherapy offers some benefits for the mental health of postpartum women. Specifically, it make improvements in maternal anxiety (SMD = −0.4, *p* = 0.002) and enhances sleep quality (SMD = −0.59, *p* = 0.002). This confirms aromatherapy as a safe, accessible non-pharmacological intervention that effectively addresses common psychological issues among mothers, offering a crucial complementary strategy particularly for women seeking to avoid medication risks.

### The effect of aromatherapy on anxiety levels in pregnant and postpartum women

Pregnancy and the postpartum period bring unique life changes—such as physical transformations, parenting stress, and health-related worries—that can easily trigger anxiety (28, 29). In evaluating the effect of aromatherapy on anxiety in this population, our meta-analysis reached a clear, positive conclusion, that is, aromatherapy confers a reduction in anxiety levels among pregnant and postpartum women. Within the central nervous system, the amygdala serves as a critical hub for processing threatening stimuli and fear responses. Its hyperactivation constitutes a core pathological basis of anxiety (30). Research indicates that inhaling essential oils rapidly modulates amygdala activity, reducing its sensitivity to potential threats and attenuating excessive sympathetic nervous system arousal. This mechanism facilitates swift alleviation of tension and hypervigilance at the neurophysiological level (31, 32). Subgroup analysis results from this study indicate that the intervention period is the primary factor influencing heterogeneity. Specifically, aromatherapy administered during the prenatal period was found to produce improvements in maternal anxiety. Therefore, it is recommended that clinical practice prioritize the application of aromatherapy during the prenatal period to effectively relieve female anxiety. Furthermore, future research should incorporate more randomized controlled trials to validate whether postpartum interventions yield therapeutic effects.

### The effect of aromatherapy on sleep quality in pregnant and postpartum women

Pregnant and postpartum women, forced to be exposed to light stimuli due to nocturia and breastfeeding, experience disrupted endogenous melatonin secretion rhythms and abnormal fluctuations in cortisol levels. Their circadian rhythms are forcibly reset, ultimately leading to poor sleep quality (33, 34). Studies indicate that aromatherapy can promote melatonin secretion by the pineal gland and reduce cortisol levels, thereby improving sleep quality (35, 36). BIARAG (37) conducted a similar meta-analysis, but this study only demonstrated that lavender essential oil and reported improvements in sleep quality. First, unlike BIARAG's focus on verifying the efficacy of a single essential oil, this study systematically incorporated and analyzed comprehensive evidence from multiple aromatherapy interventions. This robustly demonstrates that aromatherapy, as a holistic, multi-option non-pharmacological intervention strategy—rather than relying on a single component—achieves gains in sleep quality. This provides a foundation for more flexible and individualized treatment options in clinical practice. The subgroup analysis results of this study indicate that aromatherapy can effectively improve sleep quality in pregnant women. Therefore, it is recommended that aromatherapy be prioritized in clinical practice during the prenatal period to enhance sleep therapy for women. Furthermore, future research should incorporate more randomized controlled trials to validate its efficacy in postpartum interventions. Subgroup analysis by intervention period did not reveal sources of heterogeneity. However, the study demonstrated high heterogeneity, which did not significantly decrease after applying exclusion methods. Subgroup analysis by intervention timing also failed to identify sources of heterogeneity. In this regard, we believe that the inclusion of multiple intervention methods—such as inhalation, massage, and topical application—in this study may account for the observed heterogeneity in results. Potential differences in bioavailability and mechanisms of action among these delivery routes likely contribute to this variability. We recommend further exploration of these factors in future research.

## Conclusion

The findings of this study indicate that aromatherapy, as a practical, economical, and affordable non-pharmacological intervention, can effectively alleviate anxiety and improve sleep quality in pregnant women. Compared to the postpartum period, the use of aromatherapy during the prenatal period proves more effective in relieving anxiety and enhancing sleep quality. However, due to the limited number of included studies, future research should incorporate more high-quality investigations to further consolidate and validate the reliability of these conclusions.

## Research limitations

First, the included studies in this research exhibited high heterogeneity in sleep quality measures. Due to the limited number of studies, it was not possible to identify sources of heterogeneity through meta-regression or subgroup analysis. Therefore, the combined results should be interpreted with caution. Second, this study only searched English-language literature and did not include other language sources, potentially resulting in an incomplete search.

## Data Availability

The original contributions presented in the study are included in the article/Supplementary material, further inquiries can be directed to the corresponding authors.
