# Supplementary material for: The effects of aromatherapy on anxiety and sleep quality in maternal women: a systematic review and meta-analysis
Source: Front Public Health. 2025 Nov 28;13:1701126. doi: 10.3389/fpubh.2025.1701126 (PMC12698402; doi:10.3389/fpubh.2025.1701126)
Supplement: Supplementary file 1 [file Supplementary_file_1.docx]

| Database | Web of Science (all database) | |
| --- | --- | --- |
| Search Date | October 30, 2025 | |
| Search Period | From the inception of database to October 30, 2025 | |
| No. | Search strategy | Literatures retrieved |
| #1 | TS=(Pregnancy OR maternal OR pregnant OR gestational OR pregnancy) and Preprint Citation Index (Exclude – Database) | 1,678,667 |
| #2 | TS=(Postpartum Period OR postpartum OR post-pregnancy) and Preprint Citation Index (Exclude – Database) | 130,506 |
| #3 | TS=(Aromatherapy OR Aroma OR Aroma oil OR Essential oil OR Aromatherapies OR Aroma Therapy OR Aroma Therapies) and Preprint Citation Index (Exclude – Database) | 167,028 |
| #4 | TS=(Insomnia OR Sleep disorder OR Sleep wake disorders OR Sleep disturbance OR Sleep problem OR Sleep quality OR Anxiety OR Anxious OR Nervousness OR Anxiousness) and Preprint Citation Index (Exclude – Database) | 878,067 |
| #5 | #1 OR #2 | 1,705,651 |
| #6 | #3 AND #4 AND #5 | 119 |

| Database | PubMed | |
| --- | --- | --- |
| Search Date | October 30, 2025 | |
| Search Period | From the inception of database to October 30, 2025 | |
| No. | Search strategy | Literatures retrieved |
| #1 | "Pregnancy"[MeSH Terms] | 1,070,713 |
| #2 | "maternal"[Title/Abstract] OR "pregnant"[Title/Abstract] OR "gestational"[Title/Abstract] OR "pregnancy"[Title/Abstract] | 898,857 |
| #3 | "Postpartum Period"[MeSH Terms] | 82,294 |
| #4 | "postpartum"[Title/Abstract] OR "post-pregnancy"[Title/Abstract] | 91,344 |
| #5 | "Aromatherapy"[MeSH Terms] | 1,238 |
| #6 | "Aroma"[Title/Abstract] OR "aroma oil"[Title/Abstract] OR "essential oil"[Title/Abstract] OR "Aromatherapies"[Title/Abstract] OR "aroma therapy"[Title/Abstract] OR "aroma therapies"[Title/Abstract] | 34,401 |
| #7 | "Sleep Quality"[MeSH Terms] | 4,688 |
| #8 | "Insomnia"[Title/Abstract] OR "sleep disorder"[Title/Abstract] OR "sleep wake disorders"[Title/Abstract] OR "sleep disturbance"[Title/Abstract] OR "sleep problem"[Title/Abstract] | 54,178 |
| #9 | "Anxiety"[MeSH Terms] | 130,749 |
| #10 | "Anxious"[Title/Abstract] OR "Nervousness"[Title/Abstract] OR "Anxiousness"[Title/Abstract] | 25,667 |
| #11 | #1 OR #2 | 1,374,109 |
| #12 | #3 OR #4 | 145,495 |
| #13 | #5 OR #6 | 35,268 |
| #14 | #7 OR #8 | 57,780 |
| #15 | #9 OR #10 | 147,073 |
| #16 | #11 OR #12 | 1,411,517 |
| #17 | #14 OR #15 | 200,572 |
| #18 | #13 AND #16 AND #17 | 41 |

| Database | Embase | |
| --- | --- | --- |
| Search Date | October 30, 2025 | |
| Search Period | From the inception of database to October 30, 2025 | |
| No. | Search strategy | Literatures retrieved |
| #1 | 'pregnancy'/exp OR 'pregnancy' | 1,258,697 |
| #2 | 'maternal':ab,ti OR 'pregnant':ab,ti OR 'gestational':ab,ti OR 'pregnancy':ab,ti | 1,192,396 |
| #3 | 'puerperium'/exp OR 'puerperium' | 102,523 |
| #4 | 'postpartum period':ab,ti OR 'postpartum':ab,ti OR 'post-pregnancy':ab,ti | 124,271 |
| #5 | 'aromatherapy'/exp OR 'aromatherapy' | 4,991 |
| #6 | 'aroma':ab,ti OR 'aroma oil':ab,ti OR 'essential oil':ab,ti OR 'aromatherapies':ab,ti OR 'aroma therapy':ab,ti OR 'aroma therapies':ab,ti | 40,368 |
| #7 | 'sleep quality'/exp OR 'sleep quality' | 76,712 |
| #8 | 'insomnia':ab,ti OR 'sleep disorder':ab,ti OR 'sleep wake disorders':ab,ti OR 'sleep disturbance':ab,ti OR 'sleep problem':ab,ti | 89,430 |
| #9 | 'anxiety'/exp OR 'anxiety' | 643,903 |
| #10 | 'anxious':ab,ti OR 'nervousness':ab,ti OR 'anxiousness':ab,ti | 37,686 |
| #11 | #1 OR #2 | 1,588,105 |
| #12 | #3 OR #4 | 183,395 |
| #13 | #5 OR #6 | 44,124 |
| #14 | #7 OR #8 | 147,475 |
| #15 | #9 OR #10 | 655,158 |
| #16 | #14 OR #15 | 765,008 |
| #17 | #11 OR #12 | 1,653,167 |
| #18 | #17 AND #13 AND #16 | 140 |

| Database | Cochrane | |
| --- | --- | --- |
| Search Date | October 30, 2025 | |
| Search Period | From the inception of database to October 30, 2025 | |
| No. | Search strategy | Literatures retrieved |
| #1 | MeSH descriptor: [Pregnancy] explode all trees | 33882 |
| #2 | (maternal):ti,ab,kw or (pregnant):ti,ab,kw or (gestational):ti,ab,kw or (pregnancy):ti,ab,kw | 112477 |
| #3 | MeSH descriptor: [Postpartum Period] explode all trees | 2777 |
| #4 | (postpartum):ti,ab,kw or (post-pregnancy):ti,ab,kw or (post-pregnancy):ti,ab,kw or (puerperium):ti,ab,kw | 16894 |
| #5 | MeSH descriptor: [Aromatherapy] explode all trees | 444 |
| #6 | (Aroma):ti,ab,kw or (Aroma oil):ti,ab,kw or (Essential oil):ti,ab,kw or (Aromatherapies):ti,ab,kw or (Aroma Therapy):ti,ab,kw or (Aroma Therapies):ti,ab,kw | 2677 |
| #7 | MeSH descriptor: [Sleep Quality] explode all trees | 691 |
| #8 | (Insomnia):ti,ab,kw or (Sleep disorder):ti,ab,kw or (Sleep wake disorders):ti,ab,kw or (Sleep disturbance):ti,ab,kw or (Sleep problem):ti,ab,kw | 30785 |
| #9 | MeSH descriptor: [Anxiety] explode all trees | 13726 |
| #10 | (Anxious):ti,ab,kw or (Nervousness):ti,ab,kw or (Anxiousness):ti,ab,kw | 5286 |
| #11 | #1 OR #2 | 112750 |
| #12 | #3 OR #4 | 17345 |
| #13 | #11 OR #12 | 116624 |
| #14 | #5 OR #6 | 2904 |
| #15 | #7 OR #8 | 31160 |
| #16 | #9 OR #10 | 17959 |
| #17 | #15 OR #16 | 47788 |
| #18 | #13 AND #14 AND #17 | 35 |
